# Supplementary figures and images for: The C10orf76–PI4KB axis orchestrates CERT-mediated ceramide trafficking to the distal Golgi
Source: J Cell Biol. 2023 May 17;222(7):e202111069. doi: 10.1083/jcb.202111069 (PMC10192306; doi:10.1083/jcb.202111069)

**A**

anti-CERT

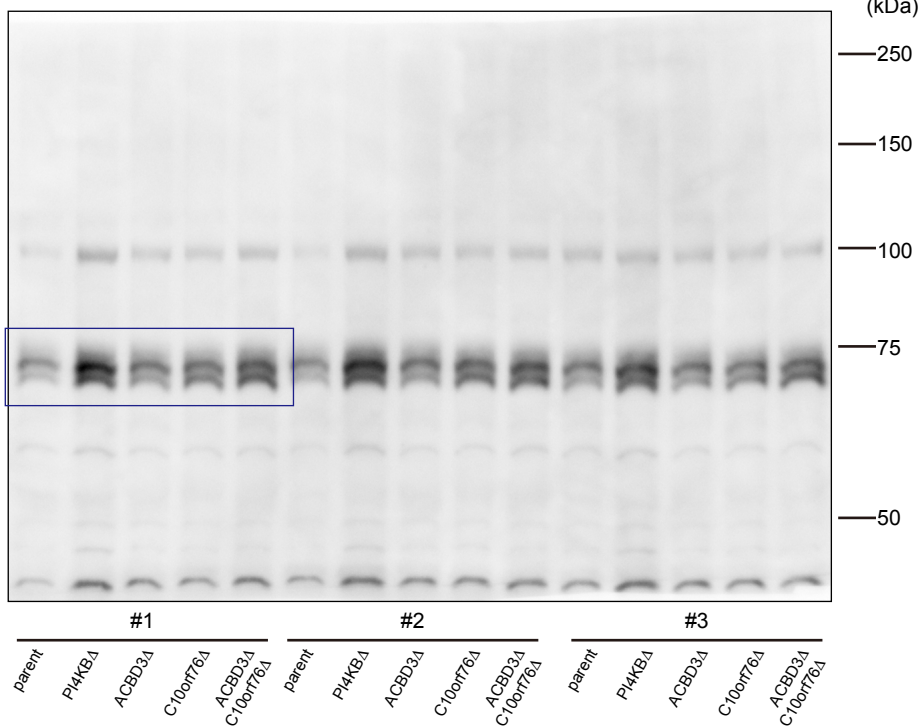

anti-GAPDH

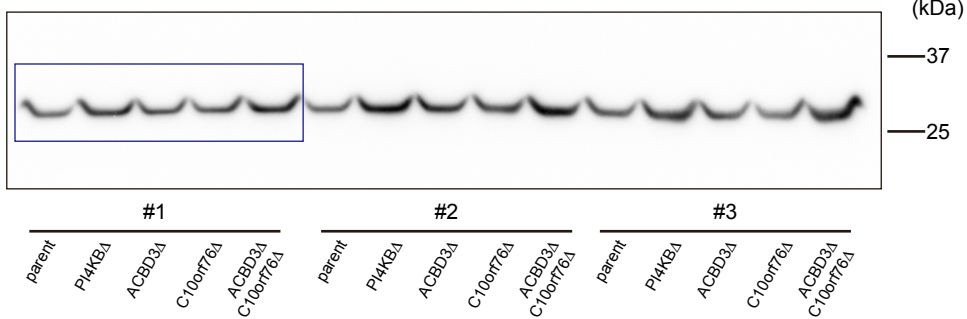

Supplement: SourceData F3 — is the source file for Fig. 3. [file JCB_202111069_SourceDataF3.pdf]

**A**

anti-PI4KB

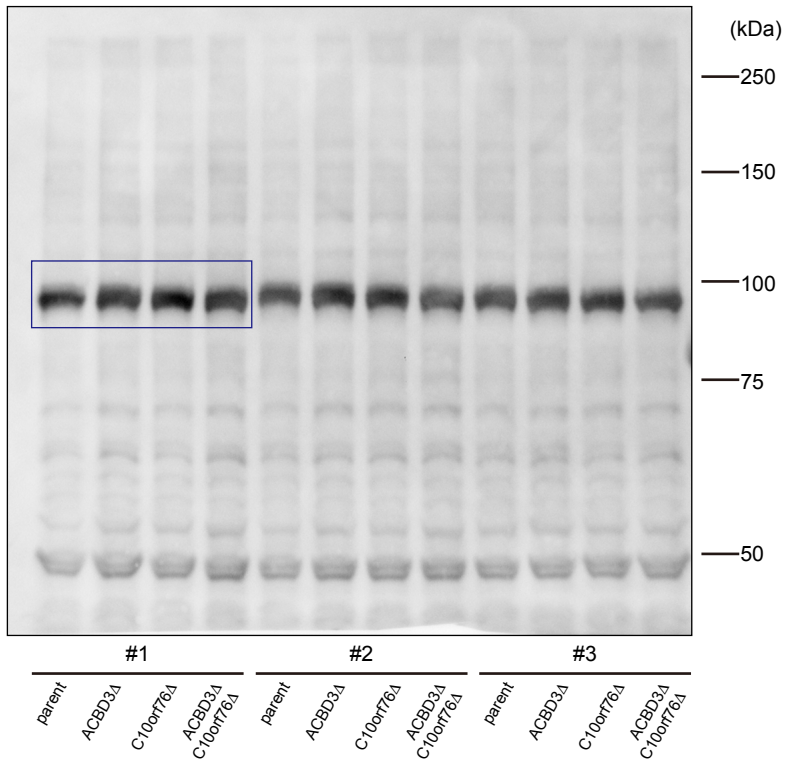

anti-GAPDH

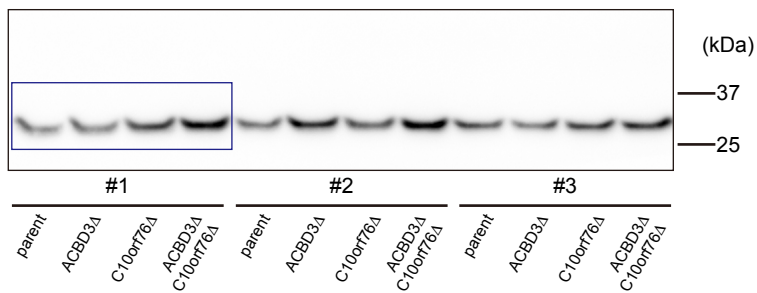

Supplement: SourceData F4 — is the source file for Fig. 4. [file JCB_202111069_SourceDataF4.pdf]

**B**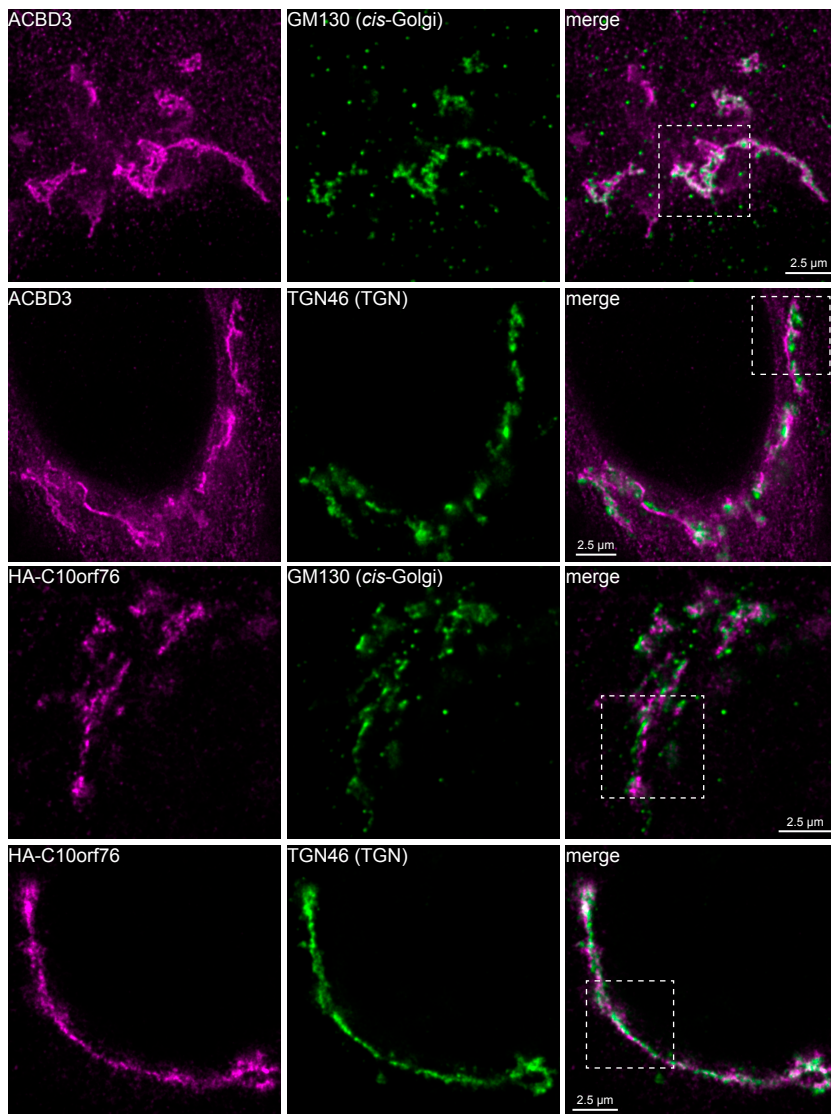**D**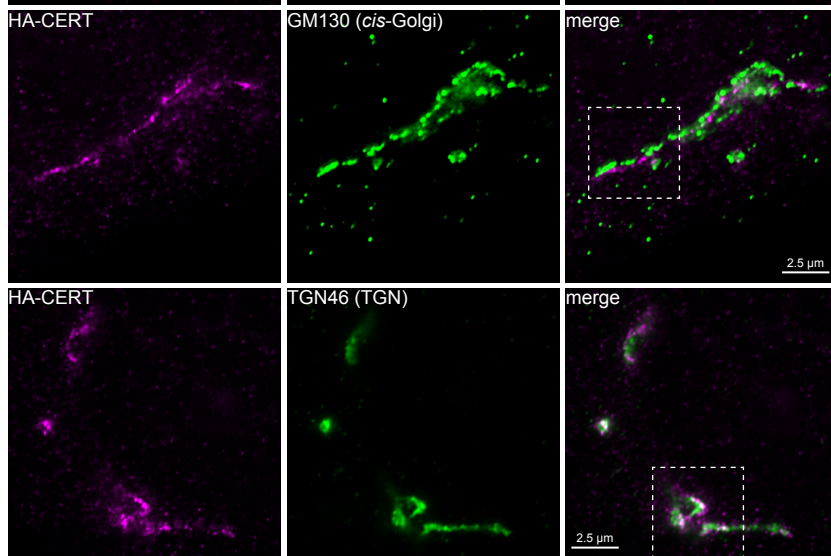

Supplement: SourceData F5 — is the source file for Fig. 5. [file JCB_202111069_SourceDataF5.pdf]

**A**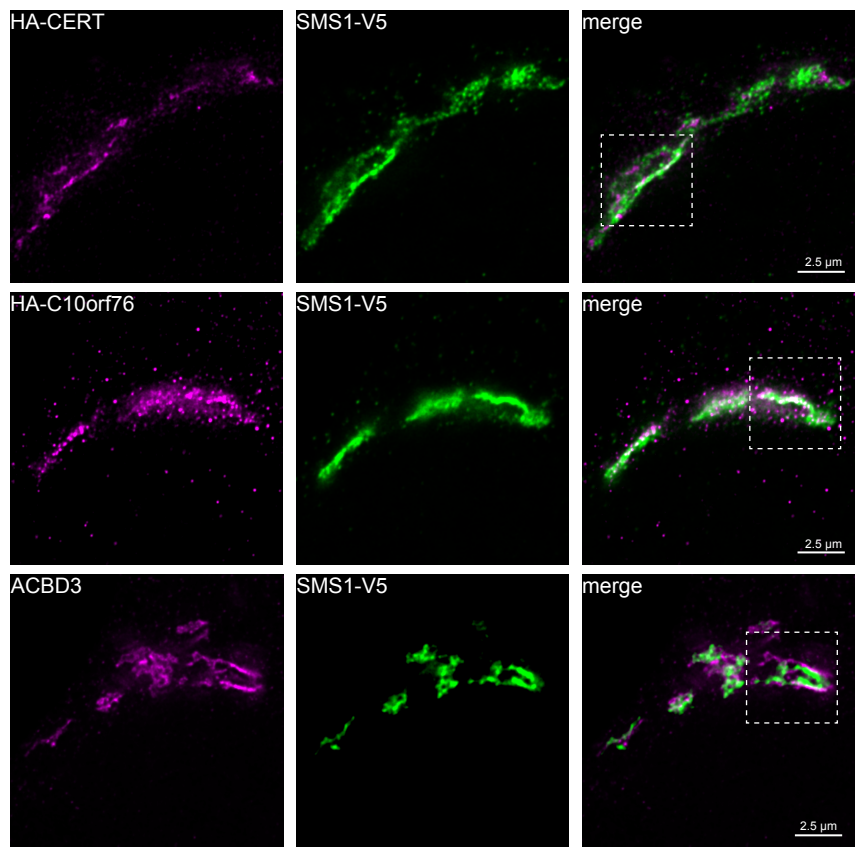**B**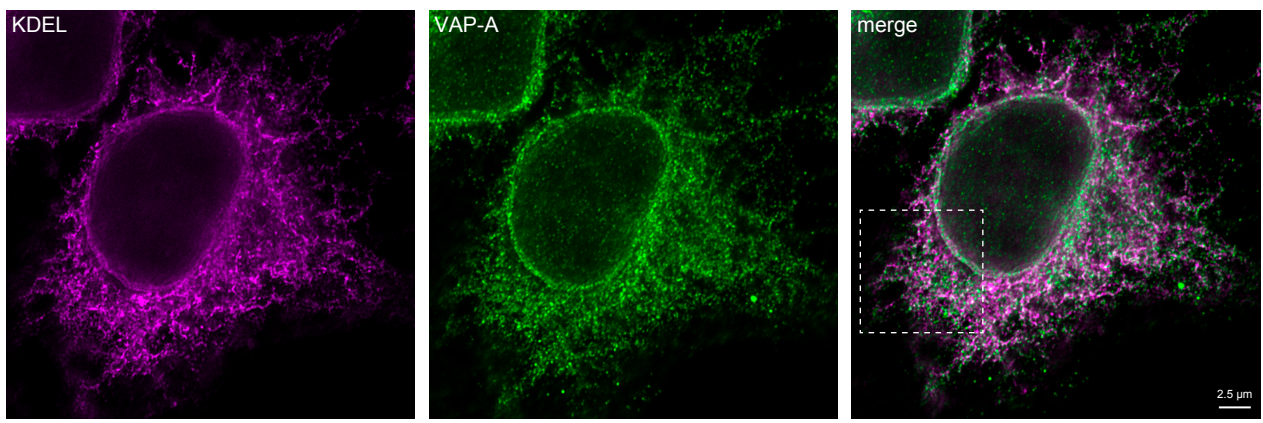**C**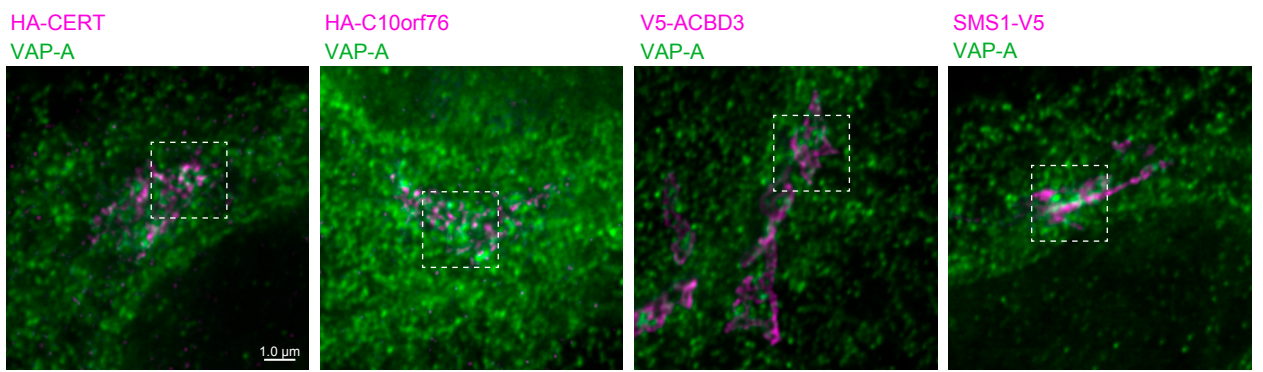

Supplement: SourceData F6 — is the source file for Fig. 6. [file JCB_202111069_SourceDataF6.pdf]

**B****anti-PI4KB**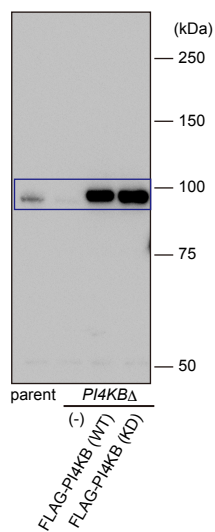**anti-ACBD3**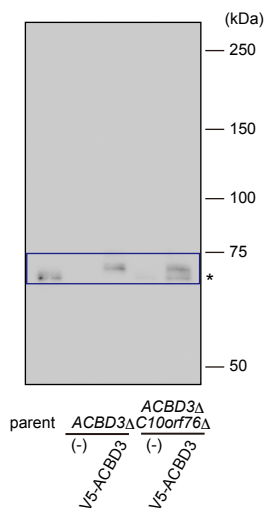**anti-C10orf76**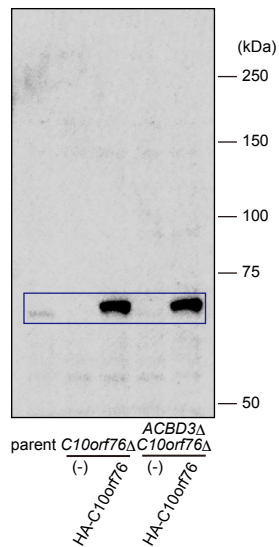**anti-GAPDH**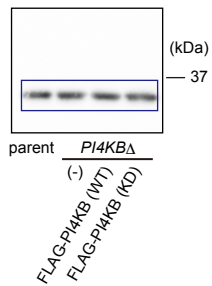**anti-GAPDH**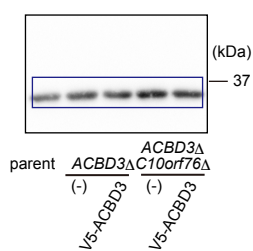**anti-GAPDH**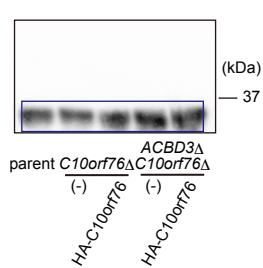

Supplement: SourceData FS2 — is the source file for Fig. S2. [file JCB_202111069_SourceDataFS2.pdf]

anti-CERT (-) myriocin

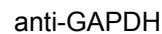

anti-CERT (+) myriocin

anti-GAPDH

# B

anti-FLAG

anti-GAPDH

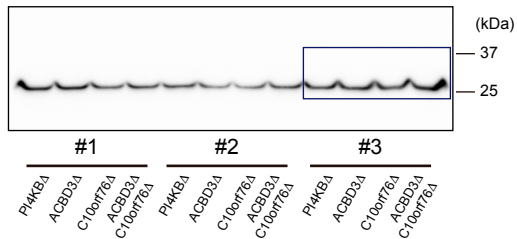

Supplement: SourceData FS3 — is the source file for Fig. S3. [file JCB_202111069_SourceDataFS3.pdf]

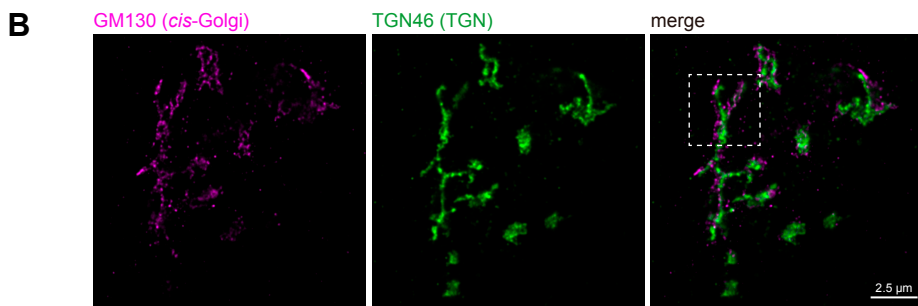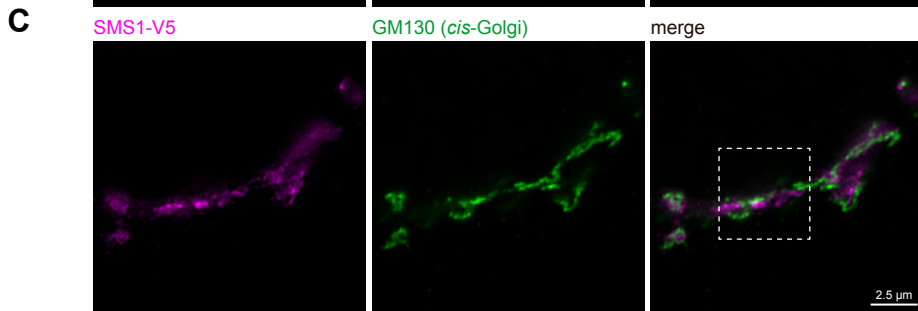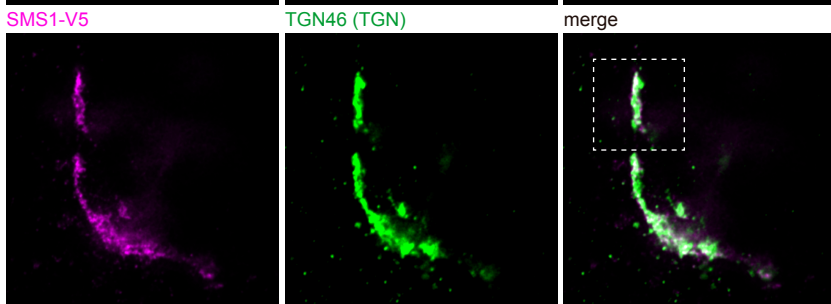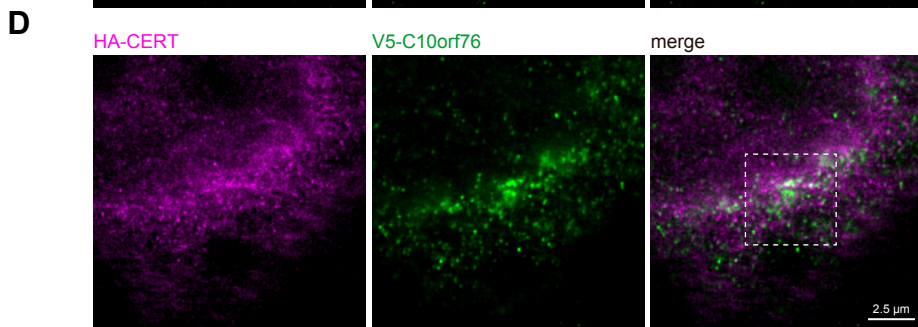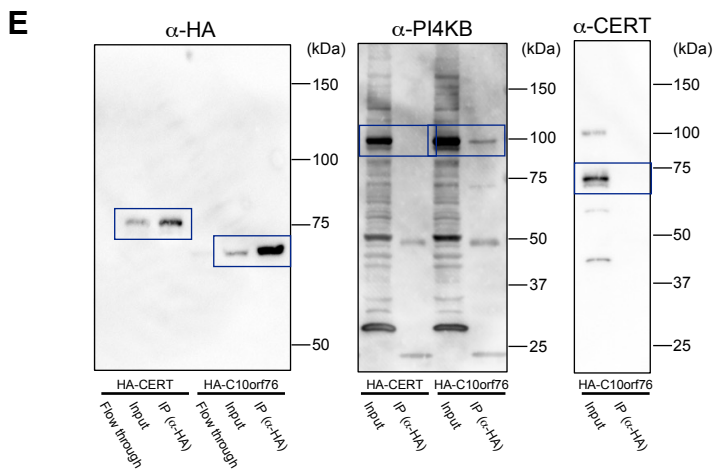

Supplement: SourceData FS4 — is the source file for Fig. S4. [file JCB_202111069_SourceDataFS4.pdf]
